# Preamble-Aided Receiver for OFDM Recovery Under Structured FMCW Interference in Co-Channel Sensing–Communication Systems

**DOI:** 10.3390/s26206540

**Published:** 2026-10-15

**Authors:** Zhiyuan Liu

**Affiliations:** School of Electrical and Electronic Engineering, Nanyang Technological University, 50 Nanyang Avenue, Singapore 639798, Singapore; zliu055@e.ntu.edu.sg

**Keywords:** integrated sensing and communication, FMCW interference, OFDM recovery, radar–communication coexistence, structured interference cancellation, preamble-aided receiver, range–Doppler processing

## Abstract

**Highlights:**

**What are the main findings?**

- A preamble-derived occupancy mask enables communication-free observations to guide both parametric FMCW reconstruction and residual-subspace compensation.
- The complete receiver achieves a 23.98 dB beat-domain cancellation gain over communication-free observations and recovers a 217-byte OFDM payload with zero BER and successful CRC verification at an input SIR of −24.30 dB.

**What are the implications of the main findings?**

- Communication-side prior information can be exploited not only for synchronization but also to constrain sensing-interference estimation and reduce the risk of fitting the desired communication signal as interference.
- Cascaded model-based and residual-subspace suppression enables end-to-end OFDM recovery under the evaluated simulation conditions.

**Abstract:**

Co-channel operation of frequency-modulated continuous-wave (FMCW) sensing and orthogonal frequency-division multiplexing (OFDM) communication can severely disrupt burst synchronization and payload recovery when both waveforms overlap in time and frequency. This paper presents a preamble-aided receiver that exploits communication-side occupancy information to constrain structured FMCW interference estimation. A known auxiliary preamble estimates the OFDM burst position and occupied temporal support, allowing communication-free observations to guide FMCW reconstruction. Dominant sensing components are estimated from range–Doppler structure and removed through regularized parametric cancellation. Residual mismatch is then suppressed using a low-dimensional subspace learned from communication-free observations and applied under the preamble-derived occupancy constraint. The compensated signal is subsequently processed by synchronization, equalization, decoding, and cyclic redundancy check (CRC) verification. In a representative partial-overlap scenario with an input communication-side signal-to-interference ratio (SIR) of −24.30 dB, the proposed receiver achieves a 23.98 dB beat-domain cancellation gain over communication-free observations, zero-sample burst-localization error, and successful recovery of a 217-byte payload with a zero bit error rate (BER) and successful CRC verification. Reduced configurations fail to recover the payload. These results provide a simulation-based proof-of-concept for communication-informed FMCW interference suppression and OFDM recovery.

## 1. Introduction

The increasing demand for simultaneous wireless sensing and communication has motivated extensive research on radar–communication coexistence and integrated sensing and communication (ISAC) systems [1,2,3,4,5]. When heterogeneous sensing and communication waveforms occupy overlapping time–frequency resources, mutual interference can arise, motivating coexistence studies involving different waveform structures [6]. More specifically, coordinated frequency-modulated continuous-wave (FMCW) and orthogonal frequency-division multiplexing (OFDM) operation has recently been investigated for ISAC [7]. On the communication side, 5G New Radio (NR) sidelink provides a relevant standardized framework for direct vehicular communication and link-level cyclic-prefix OFDM (CP-OFDM) processing [8,9,10].

Interference remains a central concern in automotive radar and ISAC systems, motivating extensive research on interference mitigation and management [11,12]. Vehicular ISAC studies further highlight the increasing coexistence of sensing and communication functions in transportation networks [13]. When coordination between sensing and communication functions is available, coexistence can be addressed through sensing–communication resource allocation [14] and joint waveform, transmit, or receive-filter design [15,16,17]. In uncoordinated or partially coordinated settings, however, the receiver must suppress the sensing component directly from the mixed observation.

Receiver-side approaches exploit several forms of signal structure. Parametric reconstruction has been applied to FMCW interference mitigation by modelling beat signals as sums of complex exponentials and estimating their parameters using matrix-pencil techniques [18]. Joint-receiver, full-duplex receiver, and interference-cancellation designs have also been investigated for ISAC systems [19,20,21]. Sparse and low-rank Hankel-matrix decomposition provides another structure-aware approach to FMCW interference mitigation [22]. Beyond explicit interference cancellation, compressed-sampling and subspace-based formulations exploit low-dimensional signal structure for joint sensing and communication processing [23,24]. These studies demonstrate the value of structure-aware processing, but they also expose an important practical issue: parametric models remain sensitive to parameter-estimation errors, leakage, weak scattering, and other model mismatch, whereas more flexible residual models may absorb part of the desired communication waveform when no additional constraint is imposed. This risk becomes particularly relevant when sensing and communication components occupy the same temporal and spectral region and only a single mixed observation is available.

Training-sequence-based methods are classical tools for OFDM timing and frequency synchronization [25,26], while Zadoff–Chu sequences have been studied for initial synchronization in LTE-like systems [27]. Transform-domain suppression provides an alternative approach for chirp-like interference; Zhang et al. [28] exploited FrFT-domain concentration to suppress chirp interference before OFDM recovery. More recent OFDM-ISAC studies have considered in-band full-duplex interference cancellation [29] and over-the-air synchronization [30]. Recent studies have also explored alternative OFDM modulation strategies [31,32] and structured discrete-time ISAC sequence design [33]. In the coexistence problem considered here, communication-side prior information can serve an additional role beyond synchronization. Specifically, an auxiliary preamble can identify both the approximate burst position and the communication-occupied temporal support. Samples outside this estimated support can therefore serve as communication-free reference observations, allowing the receiver to combine a range–Doppler structure with communication-derived occupancy information while reducing the risk of incorporating the desired OFDM waveform into the interference estimate.

Based on this idea, this work develops a preamble-aided receiver for recovering an NR-sidelink-inspired CP-OFDM burst from a single mixed baseband observation containing co-channel FMCW sensing echoes. The preamble-derived occupancy mask identifies communication-free observations that constrain both regularized parametric FMCW reconstruction and residual-subspace compensation. The compensated waveform is then returned to the pre-dechirp domain for synchronization and decoding, enabling end-to-end evaluation rather than interference-power reduction alone.

The main contributions of this work are as follows:

1. **Communication-informed interference estimation:** A preamble-aided receiver formulation is developed in which the auxiliary communication preamble is used not only for timing acquisition but also to estimate the communication-occupied temporal support. The resulting occupancy information identifies communication-free observations for FMCW estimation without requiring the true burst position, separated source signals, transmitted payload, or true sensing-path parameters.
2. **Cascaded structured interference suppression:** A two-stage suppression architecture combines regularized parametric FMCW reconstruction with residual-subspace compensation. The preamble-derived occupancy mask constrains both stages, allowing dominant range–Doppler components and structured reconstruction mismatch to be removed while reducing the risk of fitting the desired OFDM waveform as interference.
3. **Simulation-based end-to-end evaluation:** The proposed receiver is evaluated through numerical simulations using sensing, synchronization, interference-suppression, and communication metrics, including target-parameter recovery, burst-localization error, residual interference power, BER, CRC outcome, and payload recovery. In the representative partial-overlap scenario, the complete receiver achieves a 23.98 dB beat-domain cancellation gain, zero-sample burst-localization error, and successful recovery of a 217-byte payload with zero BER, whereas all reduced receiver configurations fail to recover the payload.

The remainder of this paper is organized as follows. Section 2 presents the signal model and proposed receiver architecture. Section 3 describes the simulation configuration and evaluation methodology. Section 4 reports the end-to-end recovery, ablation, and robustness results. Section 5 discusses the main findings and limitations, and Section 6 concludes the paper.

## 2. System Model and Proposed Receiver

### 2.1. Co-Channel FMCW–OFDM Signal Model

A single-channel receiver is considered in which an FMCW sensing waveform and a burst-mode CP-OFDM communication signal overlap in both time and frequency. After equivalent down-conversion and resampling, both components are represented on a common complex-baseband sampling grid with sampling interval $T_{s}$.

The transmitted FMCW waveform consists of $M_{F}$ consecutive linear frequency-modulated chirps with chirp repetition interval $T_{r}$,

(1) $$s_{F,tx}\left(t\right)=\sum_{m=0}^{M_{F}-1}g\left(t-mT_{r}\right)exp\left[j2\pi \left(f_{0}\left(t-mT_{r}\right)+\frac{\mu }{2}{\left(t-mT_{r}\right)}^{2}\right)\right]$$

where $g\left(t\right)=1$ for $0\leq t<T_{c}$ and zero otherwise, $T_{c}$ is the chirp duration, $f_{0}$ is the initial baseband frequency, and μ is the chirp slope. Considering $L_{F}$ effective sensing paths, the received FMCW component is

(2) $$r_{F}\left(t\right)=\sum_{l=1}^{L_{F}}\alpha_{l}s_{F,tx}(t-\tau_{l})e^{j2\pi f_{D,l}t}$$

where $\alpha_{l}$, $\tau_{l}$, and $f_{D,l}$ denote the complex path coefficient, propagation delay, and Doppler frequency of the l-th path, respectively. Sampling at $t=nT_{s}$ gives the discrete-time sensing component $r_{F}\left[n\right]$.

The communication waveform is an NR-sidelink-inspired CP-OFDM burst comprising a known auxiliary preamble p[n] of length $N_{p}$, followed by a payload segment $x_{data}\left[n\right]$,

(3) $$s_{C}\left[n\right]=\left\{\begin{array}{c} p\left[n\right]\\ x_{data}\left[n-N_{p}\right]\\ 0 \end{array},\;\;\;\;\;\;\;\;\begin{array}{c} 0\leq n<N_{p}\\ N_{p}\leq n<N_{p}+N_{data}\\ otherwise \end{array}\right.$$

After insertion at an unknown starting sample $n_{0}$ and propagation through a multipath channel, the received communication component is

(4) $$r_{C}\left[n\right]=\sum_{q=0}^{L_{C}-1}h_{q}s_{C}\left[n-n_{0}-d_{q}\right]$$

where $h_{q}$ and $d_{q}$ are the complex coefficient and discrete delay of the q-th path. The receiver observes only the mixed signal

(5) $$y[n]=r_{F}[n]+\alpha_{C}r_{C}[n]+v[n],v[n]∼CN(0,\sigma_{v}^{2})$$

where $\alpha_{C}$ controls the relative received communication power. The separated sensing and communication components are retained only for offline performance evaluation and are not available to the receiver during signal recovery.

The receiver is assumed to know the locally configured FMCW waveform parameters and the corresponding unit-magnitude de-chirping reference $s_{ref}\left[n\right]$, together with the auxiliary preamble, CP-OFDM numerology, burst length, resource allocation, modulation format, and decoding configuration. In contrast, the true burst position $n_{0}$, transmitted payload, separated source components, and true FMCW path parameters are unknown during recovery. The mixed signal is digitally de-chirped using

(6) $$z\left[n\right]=y\left[n\right]s_{ref}^{*}\left[n\right]$$

**Figure 1:**
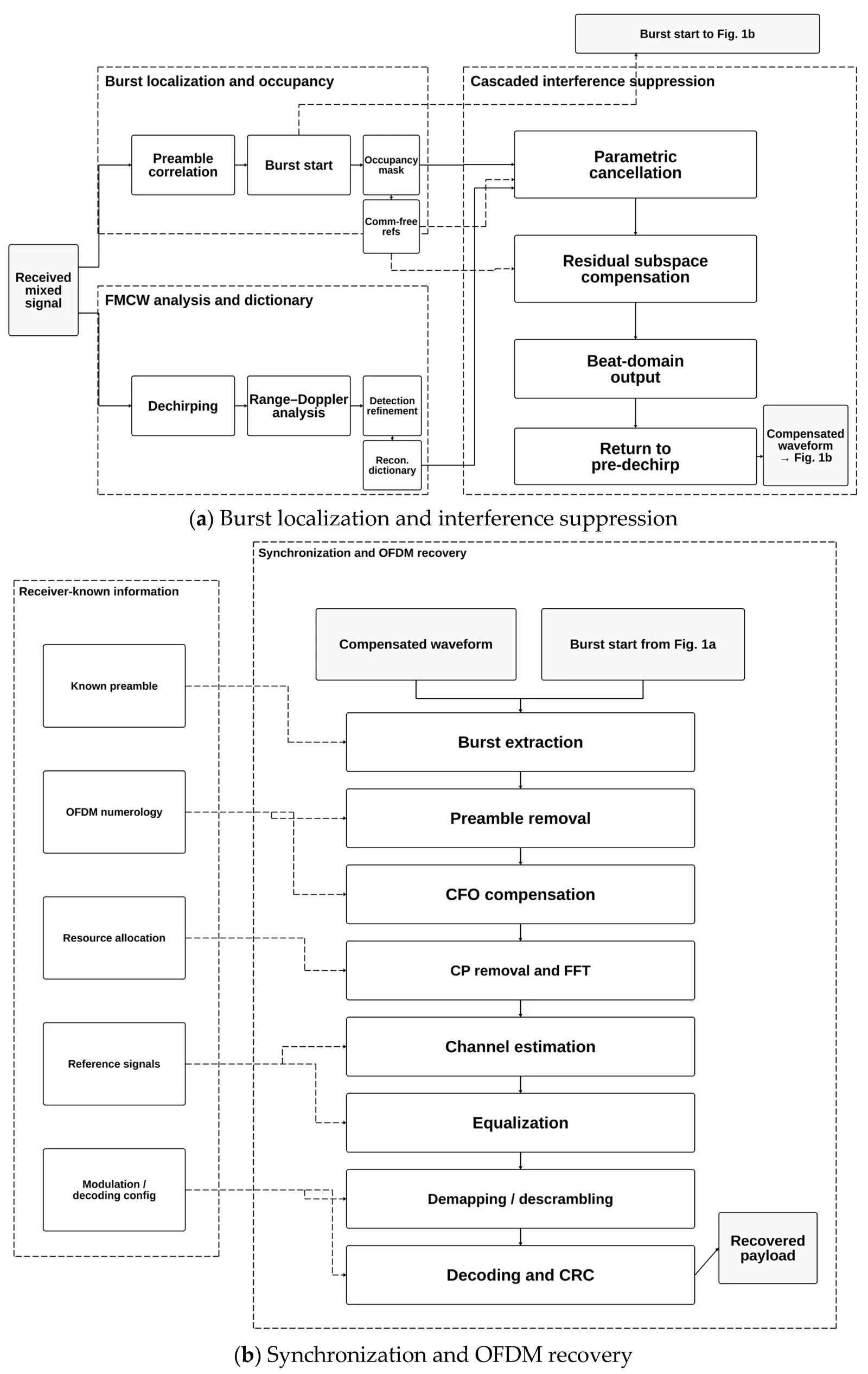
Architecture of the proposed preamble-aided receiver.

After reshaping $z\left[n\right]$ into a fast-time–slow-time representation, dominant FMCW components exhibit a localized range–Doppler structure that can be exploited for parameter estimation and reconstruction. On this basis, the proposed receiver combines communication-derived occupancy information with sensing-side structural estimation in a cascaded interference-suppression architecture. The overall processing flow is summarized in Figure 1, and the individual stages are detailed in Section 2.2, Section 2.3, Section 2.4 and Section 2.5.

### 2.2. Preamble-Aided Burst Localization and Occupancy Estimation

The known auxiliary preamble provides an initial timing reference for localizing the communication burst. Communication-channel multipath can broaden or distort the preamble-correlation response and thereby perturb the estimated burst boundary and the resulting occupancy mask. A normalized cross-correlation between the mixed observation and the preamble is evaluated as

(7) $$\rho \left[k\right]=\frac{\left|\sum_{m=0}^{N_{p}-1}y\left[k+m\right]p^{*}\left[m\right]\right|}{\sqrt{\sum_{m=0}^{N_{p}-1}{\left|y\left[k+m\right]\right|}^{2}\sum_{m=0}^{N_{p}-1}{\left|p\left[m\right]\right|}^{2}}}$$

The normalization reduces sensitivity to local amplitude variation, although strong FMCW energy can increase the normalization denominator and reduce the NCC magnitude. Its time-varying phase can also distort the correlation numerator and perturb the apparent peak position, motivating cancellation-assisted timing refinement. The initial burst-start estimate is

(8) $${\hat{n}}_{0}=\underset{k}{\mathrm{arg max}}\rho \left[k\right]$$

Given the known total burst length $N_{C}$, the estimated communication support is

(9) $${\hat{S}}_{C}=\left\{{\hat{n}}_{0},\;\ldots ,\;{\hat{n}}_{0}+N_{c}-1\right\}$$

An occupancy mask $m_{C}\left[n\right]$ is then defined as one over the estimated communication support and zero elsewhere. Samples outside ${\hat{S}}_{C}$ are treated as communication-free observations and are subsequently used to estimate FMCW interference. The purpose of this mask is not merely to indicate burst timing but to prevent samples likely to contain the desired OFDM waveform from directly contributing to sensing-interference fitting.

After de-chirping, the observation is reshaped into an $N_{F}\times M_{F}$ fast-time–slow-time matrix Z, where $N_{F}$ is the number of samples per chirp and $M_{F}$ is the number of observed chirps. Let $z_{m}$ denote the m-th chirp. Its communication-free fast-time indices are

(10) $$I_{m}=\left\{q\in \left\{0,\;\ldots ,\;N_{F}-1\right\}:m_{C}\left[mN_{F}+q\right]=0\right\}$$

A chirp is defined as a reference chirp when $\left|I_{m}\right|=N_{F}$. The set of such chirps is denoted by $C_{ref}$. Fully communication-free reference chirps are used for range–Doppler estimation and slow-time coefficient prediction, whereas the available communication-free samples within partially occupied chirps are used only for masked coefficient refinement.

It should be emphasized that $s_{ref}\left[n\right]$ in (6) denotes the locally generated FMCW waveform used for de-chirping, whereas a *reference chirp* denotes an observed chirp that lies entirely outside the estimated communication support. These two forms of reference information serve different purposes.

### 2.3. Parametric FMCW Reconstruction and Cancellation

Dominant FMCW components are first estimated from the reference chirps using windowed fast-time FFT processing, CFAR-based range detection, and slow-time Doppler FFT processing. Following range refinement and rejection of weak ghost-like detections, the retained sensing components are represented by the estimated range–velocity pairs ${\left\{\left({\hat{R}}_{k},\;{\hat{v}}_{k}\right)\right\}}_{k=1}^{K}$. The corresponding beat-domain atom for the k-th component is

(11) $$b_{k}\left[n,m\right]=exp\left(j2\pi {\hat{f}}_{b,k}nT_{s}\right)exp\left(j2\pi {\hat{f}}_{D,k}mT_{r}\right)$$

with

(12) $${\hat{f}}_{b,k}=\frac{2\mu {\hat{R}}_{k}}{c},\;{\hat{f}}_{D,k}=\frac{{2\hat{v}}_{k}}{\lambda }$$

where c is the propagation velocity and λ is the carrier wavelength.

To improve robustness against range–Doppler quantization and parameter-estimation errors, neighboring beat-frequency and Doppler atoms are introduced around each detected component. A near-range static basis is additionally included to represent leakage and low-Doppler scattering. The complete reconstruction dictionary is written as

(13) $$B=\left[B_{tar},\;B_{comp},\;B_{stat}\right]$$

where the three blocks correspond to the target-locked atoms, neighboring compensation atoms, and near-range static atoms, respectively. Their numerical configurations are specified in Section 3. Because neighboring atoms are intentionally correlated, ridge regularization is used to stabilize the coefficient estimation.

Let $B_{m}$ denote the dictionary restricted to the m-th chirp. For $m\in C_{ref}$, the coefficient vector is estimated using ridge-regularized least squares.

(14) $${\hat{a}}_{m}=\underset{a}{\arg\;\min}{\left\|z_{m}-B_{m}a\right\|}_{2}^{2}+\lambda_{r}{\left\|a\right\|}_{2}^{2}$$

Here, ridge-regularized least squares is used as a deterministic fitting procedure and does not rely on a white-noise optimality assumption. The estimated coefficients from the reference chirps are interpolated along slow time to produce a prior ${\tilde{a}}_{m}$ for chirps affected by communication overlap. Its reliability depends on retaining sufficient communication-free reference chirps around the occupied interval. A larger temporal gap increases interpolation uncertainty and may alter the optimal balance between data fitting and prior regularization.

For a partially occupied chirp, let $M_{m}$ be a diagonal mask with unit entries over $I_{m}$ and zeros elsewhere. The coefficient vector is refined as

(15) $${\hat{a}}_{m}=\underset{a}{\arg\;\min}{\left\|M_{m}\left(z_{m}-B_{m}a\right)\right\|}_{2}^{2}+\lambda_{r}{\left\|a\right\|}_{2}^{2}+\lambda_{p}{\left\|a-{\tilde{a}}_{m}\right\|}_{2}^{2}$$

Thus, samples estimated to contain the communication burst do not contribute to the data-fitting term. If a chirp is fully occupied, masked refinement is skipped and the interpolated prior is used directly. The residual after parametric cancellation is

(16) $$z_{res,m}=z_{m}-B_{m}{\hat{a}}_{m}$$

This stage removes the dominant physically interpretable FMCW structure. Residual correlated mismatch caused by imperfect parameter estimation, weak scattering, leakage, and other unmodelled components is addressed by the second suppression stage.

### 2.4. Mask-Constrained Residual-Subspace Compensation

Residual vectors from the reference chirps are assembled as columns of $E_{ref}\in C^{N_{F}\times N_{ref}}$, where $N_{ref}=\left|C_{ref}\right|$. After removing the mean across the reference-chirp dimension, the centered residual matrix is decomposed using singular value decomposition,

(17) $$E_{c}=U\Sigma V^{H}$$

The residual subspace $U_{sub}$ is formed from the leading left singular vectors. Its dimension is determined using a cumulative-energy criterion together with a maximum-rank constraint; the numerical settings are provided in Section 3.

Residual-subspace coefficients on the reference chirps are first obtained by projection onto $U_{sub}$, and then interpolated along slow time to form priors for the remaining chirps. For partially occupied chirps, the coefficients are refined using the same mask-constrained and prior-regularized principle as in (15), with the parametric dictionary replaced by the residual basis. Consequently, only communication-free samples contribute directly to the residual fitting. For fully occupied chirps, the interpolated prior is used without further fitting. The compensated beat-domain signal is

(18) $$z_{comp,m}=z_{res,m}-U_{sub}{\hat{c}}_{m}$$

After all chirps are concatenated, the compensated sequence is returned to the pre-dechirp complex-baseband domain through multiplication by $s_{ref}\left[n\right]$.

As summarized in Figure 1, the two suppression stages therefore have complementary roles. The parametric stage removes dominant components whose range–Doppler structure can be explicitly estimated, whereas the residual-subspace stage represents structured mismatch not captured by the parametric model. Importantly, both stages are constrained using communication-derived occupancy information.

### 2.5. Synchronization and OFDM Recovery

After interference suppression, the recovered pre-dechirp waveform is passed to the communication receiver for burst extraction and decoding. The preamble-derived burst-start estimate provides the timing reference for extracting the communication interval.

Following burst extraction, the auxiliary preamble is removed, and the remaining signal is processed by the CP-OFDM receiver. Because cancellation is constrained by the estimated communication occupancy, communication-bearing samples do not directly drive the interference fit, reducing the risk of degrading CP-based synchronization. Residual modelling error may nevertheless perturb the CP correlation; therefore, residual CFO is estimated and compensated from the cyclic prefix after interference suppression. The cyclic prefixes are then removed and FFT demodulation is performed. Known reference signals are used for residual phase correction and channel estimation before equalization, demapping, descrambling, rate recovery, channel decoding, CRC verification, and payload parsing.

The complete processing chain therefore enables evaluation in terms of successful end-to-end communication recovery rather than interference-power reduction alone.

## 3. Simulation and Evaluation Setup

### 3.1. Waveform and Co-Channel Scenario

The FMCW sensing waveform uses a 77 GHz carrier frequency, a 150 MHz sweep bandwidth, and a 40 μs chirp duration, corresponding to a chirp slope of 3.75 THz/s. Each sensing frame contains 128 chirps with 512 native samples per chirp at a sampling rate of 12.8 MHz. Three dominant targets are located at $\left(50\;m,18\;m/s\right)$, $\left(32\;m,-10\;m/s\right)$, and $\left(71\;m,0\;m/s\right)$, with relative amplitudes of 1.00, 0.65, and 0.40, respectively.

To introduce mismatch between the received sensing signal and the parametric reconstruction model, the simulated FMCW component additionally contains 20 weak clutter scatterers distributed over 5–120 m, a leakage component with a relative amplitude of 1.6 at an equivalent range of 1 m, 200 ppm chirp-slope drift, 30 ps timing jitter, a 50 Hz phase-noise linewidth, sweep nonlinearity, and additive sensing-side disturbance. The additive disturbance consists of white and colored Gaussian components. The white component is scaled using a 35 dB reference SNR, while the colored component is generated using a first-order autoregressive model with a coefficient of 0.995 and a driving-noise power equal to 5% of that of the white component. These impairments are included in sensing-signal generation but are not explicitly represented by the dominant-component reconstruction model, thereby introducing structured model mismatch for evaluating the second-stage compensation. This sensing-side disturbance is fixed throughout the subsequent receiver-noise sweep and is treated as part of the FMCW model mismatch rather than as the receiver-noise term v[n] in (5).

The communication waveform follows an NR-sidelink-inspired CP-OFDM configuration with a 20 MHz bandwidth template, 30 kHz subcarrier spacing, 16-QAM modulation, and 20 allocated physical resource blocks. Two slots form a 30,720-sample payload, preceded by a 127-sample Zadoff–Chu auxiliary preamble. The total communication-burst length is therefore 30,847 samples.

Before signal superposition, the FMCW waveform is resampled to the 30.72 MHz communication sampling rate, yielding approximately 1229 samples per chirp and 157,312 sensing samples in total. In the representative partial-overlap realization, the communication burst begins at sample $n_{0}=40,957$. This true position is not provided to the receiver and is retained only for offline timing-error evaluation.

The identity channel is used as the controlled reference condition to isolate structured FMCW interference from communication-channel distortion. To assess whether the receiver behavior is specific to this idealized case, an additional static three-tap frequency-selective communication channel is evaluated. The tap delays are 0, 4, and 9 samples, with relative gains of 0, −6, and −12 dB, respectively. The maximum excess delay of 9 samples remains well within the minimum OFDM cyclic prefix of 72 samples. The communication-burst power is normalized to that of the identity-channel case, while all receiver parameters remain unchanged.

### 3.2. Receiver Configuration and Algorithm Parameters

Unless otherwise stated, the mixed observation is processed at a sampling rate of 30.72 MHz with zero nominal communication carrier-frequency offset. Independent complex additive white Gaussian noise (AWGN) is added at the receiver input. Its variance is adjusted such that, after de-chirping and beat-domain low-pass filtering, the resulting noise power is −45 dBFS relative to the analog-to-digital converter (ADC) full-scale power. Since the de-chirping reference has unit magnitude, de-chirping itself preserves the noise power, while the pre-filter noise variance compensates for the energy response of the beat-domain filter. The beat-domain observation is filtered using a 161-tap finite-impulse-response (FIR) low-pass filter (LPF) with a cutoff frequency of 3.463 MHz and is subsequently quantized to 12 bits without clipping. Because the receiver is modeled in the complex electrical-baseband domain, thermal and receiver noise are represented by Gaussian processes; Poisson counting statistics are outside this model.

All receiver parameters are fixed across the reported simulations. Range and Doppler processing use FFT sizes of 4096 and 512, respectively. The one-dimensional constant false-alarm rate (CFAR) detector uses 20 training bins and 3 guard bins on each side of the cell under test, with a threshold factor of 6.5 relative to the mean training-cell power. Adjacent detections separated by no more than three range bins are merged and locally refined within ±1 range bin, with at most eight components retained. Components lying within 0.5 m/s of a stronger detection and at least 12 dB lower in power are rejected.

Each retained target component is augmented using range-bin offsets $\left\{-2,-1,0,1,2\right\}$ and Doppler-bin offsets $\left\{-1,0,1\right\}$. The near-range static basis spans 0–6 m with a spacing of 0.30 m and uses the same Doppler-bin offsets.

Regularization parameters are set to $\lambda_{r}=10^{-6}$ and $\lambda_{p}=5\times 10^{-4}$. These values are fixed across all experiments and provide mild stabilization of the correlated dictionary without retuning between operating points. No gap-dependent retuning of $\lambda_{p}$ or $\lambda_{r}$ is performed. The complex coefficient trajectories are first smoothed over five chirps and then interpolated using piecewise cubic Hermite interpolation (PCHIP). The residual-subspace dimension is selected using a target cumulative singular-value energy of 0.985, an upper cumulative-energy limit of 0.995, a relative singular-value threshold of 0.012, and a maximum rank of 12.

### 3.3. Ablation Configurations and Evaluation Metrics

To isolate the contribution of the successive receiver stages, four nested receiver configurations are evaluated using the same mixed-signal realization and the same downstream communication-processing chain.

The direct mixed-signal receiver performs OFDM recovery without FMCW interference cancellation. The dominant-target reconstruction receiver removes only the detected target-locked range–Doppler components. The parametric FMCW cancellation receiver additionally employs the augmented reconstruction dictionary introduced in Section 2.3, including the neighboring compensation atoms and near-range static basis but does not apply residual-subspace compensation. The full proposed receiver further applies the mask-constrained residual-subspace stage before synchronization and OFDM recovery. These configurations form an ablation study designed to determine whether each successive interference-suppression stage is necessary for successful end-to-end payload recovery.

For communication-side characterization, the input signal-to-interference ratio is evaluated offline over the active communication interval $\Omega_{c}$ as

(19) $${SIR}_{in}=10{log}_{10}\frac{\sum_{n\in \Omega_{c}}{\left|x_{C}\left[n\right]\right|}^{2}}{\sum_{n\in \Omega_{c}}{\left|x_{F}\left[n\right]\right|}^{2}}$$

where $x_{C}\left[n\right]=\alpha_{C}r_{C}\left[n\right]$ and $x_{F}\left[n\right]=r_{F}\left[n\right]$ denote the received communication and FMCW components as they appear in the mixed observation. These separated components are retained only for offline evaluation and are not available to the receiver. At the representative operating point, ${SIR}_{in}=-24.30\;dB$. Burst-localization accuracy is quantified by

(20) $$e_{tim}={\hat{n}}_{b}-n_{0}$$

where ${\hat{n}}_{b}$ denotes the final burst-start estimate produced by the receiver configuration under evaluation, and $n_{0}$ is retained only as offline ground truth. Sensing recovery is characterized by the range and velocity errors of the detected dominant components.

Residual suppression is evaluated in the beat domain over the estimated communication-free sample set $\Omega_{free}$. Let $z_{pre}\left[n\right]$ denote the original beat-domain observation before any interference cancellation, and let $z_{post}\left[n\right]$ denote the beat-domain output of the receiver configuration under evaluation. The residual power ratio is defined as

(21) $$\eta_{res}=\frac{\sum_{n\in \Omega_{free}}{\left|z_{post}\left[n\right]\right|}^{2}}{\sum_{n\in \Omega_{free}}{\left|z_{pre}\left[n\right]\right|}^{2}}$$

The same pre-cancellation observation $z_{pre}\left[n\right]$ is used as the reference for all receiver configurations, and the corresponding beat-domain cancellation gain is

(22) $$G_{can}=-10{log}_{10}(\eta_{res})$$

Because this metric is evaluated only over the estimated communication-free region, it characterizes suppression of the structured FMCW component without direct contamination from the desired OFDM burst. End-to-end communication performance is evaluated using burst-localization error, CRC outcome, payload recovery, and BER when a valid payload decode is obtained. An external FrFT-based baseline adapted from [28] is also evaluated using the same mixed observation and downstream communication-processing chain. The FrFT order is selected by a coarse-to-fine search using the known local FMCW reference, yielding (a=−0.128). Samples exceeding ($\mu_{\left|Y\right|}+4\sigma_{\left|Y\right|}$) in the selected FrFT domain are set to zero before inverse transformation. The parameters are fixed for the comparison. For method-independent suppression evaluation, all external methods are additionally scored over the same offline reference interval comprising the complete FMCW chirps preceding the true communication burst (chirps 1–33); the true burst position is used only for this offline metric and is not provided to any receiver.

### 3.4. Robustness and Random-Instance Protocol

Communication contamination of the sensing-processing branch is characterized using the post-LPF beat-domain interference-to-noise ratio (INR), defined as the communication-component power relative to the receiver-noise power after de-chirping and low-pass filtering. The representative operating point uses a beat-domain INR of 10 dB. This quantity characterizes communication leakage into the FMCW processing branch and is distinct from the communication-side FMCW-to-OFDM interference condition represented by ${SIR}_{in}$.

Robustness is evaluated using two parameter sweeps, while all receiver parameters remain fixed. In the receiver-noise sweep, the communication amplitude is fixed, while the post-LPF receiver-noise level is varied from −45 to −25 dBFS, causing the beat-domain INR to decrease from 10 to −10 dB. In the beat-domain INR sweep, the receiver-noise level is fixed at −45 dBFS, while the communication amplitude is adjusted to obtain INR values from 10 to 30 dB. No receiver parameter is retuned between sweep points.

Random-instance stability is further evaluated using 10 independent receiver-noise realizations at the representative operating point. Only the random noise seed is varied, while the sensing scene, communication waveform, overlap position, and receiver configuration remain unchanged. Timing accuracy, payload decoding, BER, and CRC verification are recorded for each realization.

### 3.5. Computational Complexity

The computational cost is dominated by range–Doppler processing, ridge-regularized coefficient estimation, and residual-subspace learning. Let N denote the number of fast-time samples per chirp, M the number of chirps, K the reconstruction-dictionary size, $M_{r}$ the number of communication-free reference chirps, and r the retained residual-subspace rank. The range and Doppler FFT stages require $O(MN_{R}log\;N_{R})$ and $O(MN_{D}log\;N_{D})$ operations, respectively. Ridge-regularized least-squares estimation requires $O(M(NK^{2}+K^{3}))$, while economy-size SVD of the $N\times \;M_{r}$ residual matrix requires $O(NM_{r}^{2})$ for $N\geq M_{r}$, followed by O(rNM) low-rank projection and compensation.

In the representative configuration, N≈1229, M=128, $N_{R}=4096$, and $N_{D}=512$, the reconstruction dictionary contains 108 atoms, and the residual rank is limited to 12. The current MATLAB R2025b implementation is intended as a proof-of-concept and is not used to claim a specific real-time latency. The FFT and bounded-dimension linear-algebra operations are amenable to parallel or hardware-accelerated implementation.

## 4. Simulation Results and Analysis

### 4.1. Representative End-to-End Recovery

The representative partial-overlap scenario is first used to examine the complete receiver chain under severe co-channel interference. At this operating point, the communication-side input SIR is −24.30 dB and the post-LPF beat-domain INR is 10 dB.

**Figure 2:**
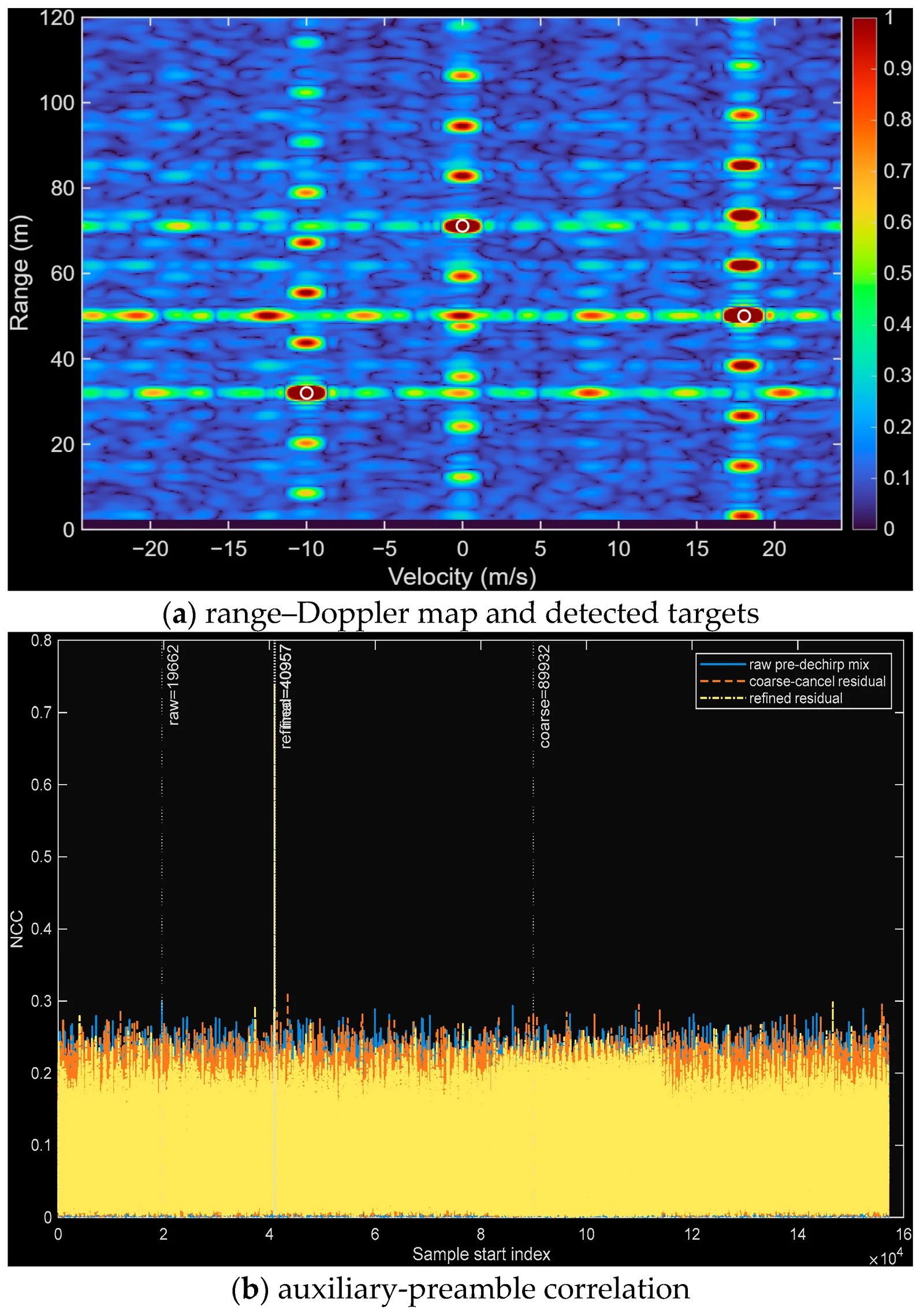
Representative recovery results.

The preamble-derived communication-free region provides sufficient reference observations for estimating the dominant FMCW components. As shown in Figure 2a, all three simulated targets are correctly detected. The mean absolute range and velocity errors are 0.067 m and 0.026 m/s, respectively, indicating that the dominant sensing structure remains identifiable in the mixed observation.

Interference suppression also substantially improves the visibility of the auxiliary preamble. Figure 2b compares the normalized preamble-correlation profiles before and after cancellation. The peak correlation increases from 0.2978 in the original mixed observation to 0.7679 after interference suppression. The estimated burst start coincides with the true starting sample $n_{0}=40,957$, corresponding to $e_{tim}=0$.

**Figure 3:**
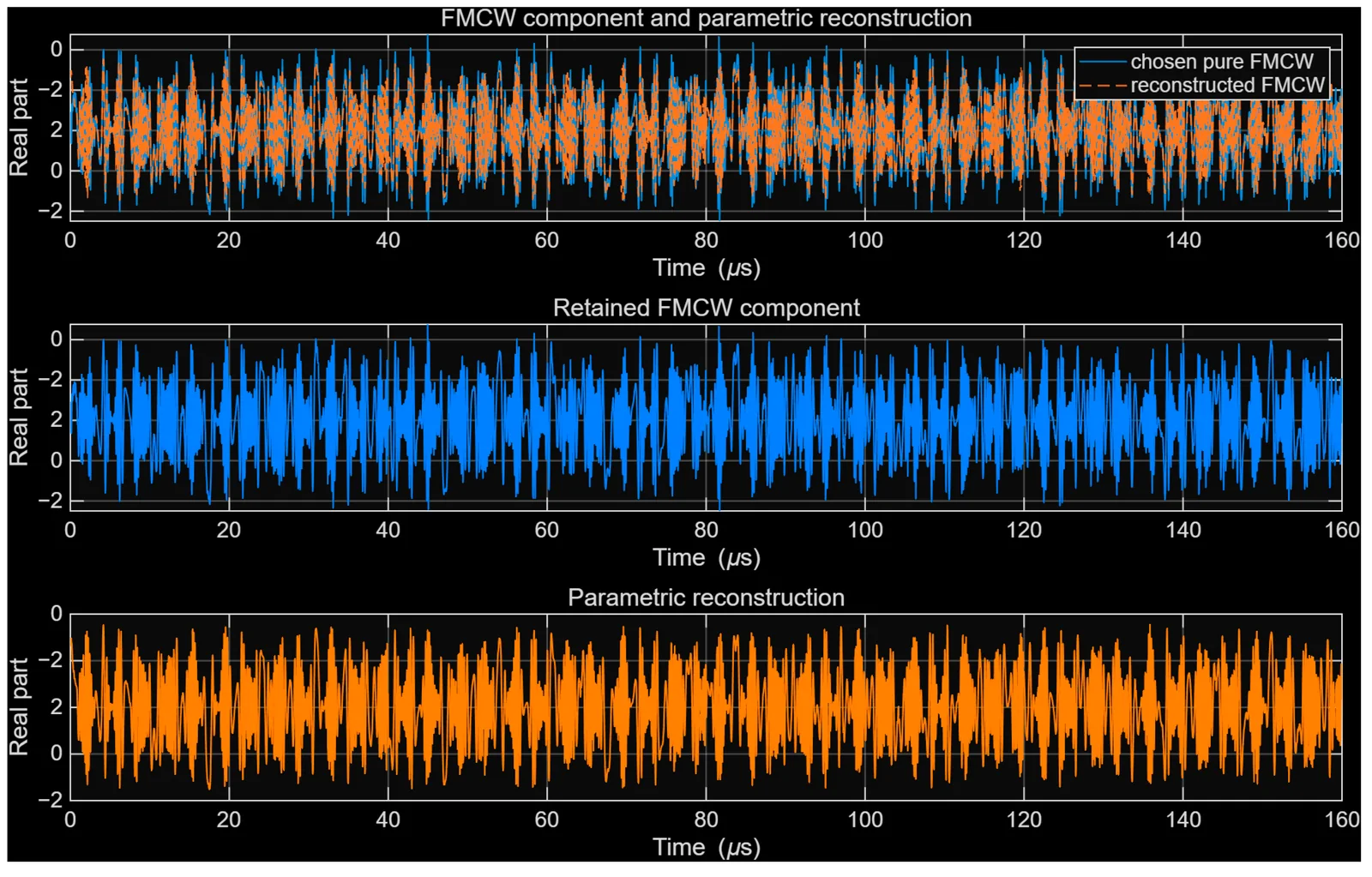
FMCW component and reconstruction before subspace compensation.

The parametric reconstruction captures the dominant FMCW waveform structure but does not reproduce the received sensing component exactly. As illustrated in Figure 3, residual correlated mismatch remains because weak clutter, leakage, chirp-slope drift, timing jitter, phase noise, and sweep nonlinearity are not explicitly represented by the dominant parametric model. This residual structure is consistent with the ablation result: parametric cancellation provides 8.06 dB gain, increasing to 23.98 dB after residual-subspace compensation.

After residual-subspace compensation, synchronization, and CP-OFDM processing, the complete 30,720-sample communication payload is successfully recovered. The decoded 217-byte transport block passes CRC verification and matches the transmitted payload, resulting in zero payload BER. Thus, successful recovery is achieved despite the representative communication-side input SIR of −24.30 dB.

### 4.2. Ablation of the Cascaded Suppression Stages

The contribution of the successive interference-suppression stages is evaluated using the four nested receiver configurations defined in Section 3.3. Table 1 summarizes the corresponding residual suppression and end-to-end communication outcomes.

The direct mixed-signal receiver produces a burst-localization error of $e_{tim}=-4089$ samples and fails CRC verification. Dominant-target reconstruction reduces the residual power ratio to 0.1780, corresponding to a 7.50 dB cancellation gain, but still fails decoding. The augmented parametric dictionary further reduces the residual power ratio to 0.1564 (8.06 dB), indicating that parametric expansion alone does not adequately capture the remaining structured mismatch.

The full receiver reduces the residual power ratio to 0.0040, corresponding to a 23.98 dB cancellation gain. Relative to the parametric-only configuration, residual-subspace compensation provides 15.92 dB of additional suppression and yields $e_{tim}=0$, successful CRC verification, and zero payload BER. It is the only evaluated configuration that successfully recovers the transmitted payload.

### 4.3. Comparison with an External FrFT Baseline

Table 2 compares the proposed receiver with the adapted FrFT baseline under the representative mixed-signal realization. For this external comparison, suppression gain is evaluated over the same leading communication-free reference chirps for both methods; this reference-region metric is distinct from the ablation metric $G_{can}$ reported in Table 1.

The FrFT baseline achieves 2.78 dB of suppression but fails to localize and decode the communication burst. In contrast, the proposed receiver achieves 24.92 dB over the same reference interval, zero-sample timing error, and successful recovery of the 217-byte payload with zero BER and valid CRC. As an additional diagnostic, FrFT processing also fails to decode the payload when the true burst position is supplied offline, indicating that its failure is not attributable solely to burst-localization error.

### 4.4. Robustness and Random-Instance Stability

The robustness sweeps are summarized in Table 3. Burst localization remains exact in all tested cases. The receiver-noise sweep yields zero BER and valid CRC throughout, with an approximately constant cancellation gain of 24.38 dB. In contrast, increasing beat-domain INR reduces the cancellation gain from 24.38 to 13.25 dB, and decoding fails at INR values of 20 dB and above.

The beat-domain INR represents communication contamination of the FMCW sensing-processing branch after de-chirping and low-pass filtering and should not be interpreted as a conventional communication-side SINR. It therefore characterizes the sensitivity of the interference-estimation and recovery chain to increasing communication leakage into the sensing-processing branch.

Random-instance stability is further evaluated over 10 independent receiver-noise realizations at the representative operating point. All 10 realizations achieve zero-sample localization error, zero payload BER, valid CRC, and correct recovery of the 217-byte payload, demonstrating repeatability with respect to receiver-noise realizations rather than statistical robustness over arbitrary channels or sensing scenes.

A three-tap frequency-selective communication channel is additionally evaluated as specified in Section 3.1. As shown in Table 4, zero-sample localization error and valid CRC recovery are retained, with a residual power ratio of 0.0038 and a 24.20 dB cancellation gain. Thus, end-to-end recovery is not restricted to the identity-channel reference case.

## 5. Discussion

The ablation study indicates that the two suppression stages serve complementary functions. Parametric reconstruction captures the dominant range–Doppler structure, whereas residual-subspace compensation addresses structured mismatch that remains after model-based cancellation. The additional 15.92 dB suppression provided by the second stage therefore reflects the importance of modelling residual interference that cannot be represented adequately by the dominant parametric basis.

The communication-derived occupancy mask is central to this architecture. By identifying samples unlikely to contain the desired OFDM waveform, it provides reference observations for both parametric estimation and residual-subspace learning. This reduces the risk that communication-bearing samples directly influence the interference estimate and links synchronization information with interference suppression rather than treating the two tasks independently. The external FrFT comparison further shows that exploiting chirp concentration alone provides measurable interference suppression but is insufficient for end-to-end recovery in the evaluated severe-interference condition. The substantially larger suppression and successful decoding obtained by the proposed receiver support the use of communication-derived occupancy information to constrain interference estimation.

The robustness results further suggest that burst localization is not the primary performance bottleneck. Degradation in end-to-end recovery occurs after acquisition remains reliable, indicating that the limiting factors lie in the subsequent interference reconstruction, synchronization, equalization, and decoding stages. The random-noise trials support repeatability at the representative operating point, but they do not establish statistical robustness over broader channel or sensing conditions.

Several limitations define the scope of the present study. The receiver relies on sufficient communication-free observations for sensing-interference estimation, so larger overlap fractions may reduce the available reference data. Although an additional static frequency-selective multipath case is evaluated, broader propagation conditions such as larger delay spreads and time-varying channels remain to be investigated. The auxiliary preamble also introduces signaling overhead. The present study is therefore limited to a simulation-based proof-of-concept without hardware or over-the-air validation.

## 6. Conclusions

This work presented a preamble-aided receiver for OFDM recovery under co-channel FMCW sensing interference. A communication-derived occupancy mask is used to identify communication-free observations and constrain both regularized parametric FMCW reconstruction and residual-subspace compensation, after which the compensated waveform undergoes synchronization and OFDM decoding.

In the representative partial-overlap scenario with a communication-side input SIR of −24.30 dB, the complete receiver achieves a 23.98 dB beat-domain cancellation gain, zero-sample burst-localization error, and successful recovery of a 217-byte payload with zero BER and successful CRC verification. The ablation results show that parametric cancellation alone is insufficient and that residual-subspace compensation is required for successful end-to-end recovery. The external baseline and robustness tests, including the three-tap multipath case, further support the proposed communication-informed suppression approach under the evaluated conditions. Together, these findings provide a simulation-based proof-of-concept for the proposed receiver. Further hardware and over-the-air validation under realistic propagation and RF conditions is needed to assess practical applicability.

## Figures and Tables

**Table 1:** Ablation results across receiver configurations.

Receiver Configuration	$e_{tim}$ (Samples)	$\eta_{res}$	$G_{can}$ (dB)	CRC
Direct mixed-signal receiver	−4089	1.0000	0.00	Fail
Dominant-target reconstruction	17,012	0.1780	7.50	Fail
Parametric FMCW cancellation	−1200	0.1564	8.06	Fail
Full proposed receiver	0	0.0040	23.98	Pass

**Table 2:** Comparison with the external FrFT-based baseline.

Receiver Configuration	$e_{tim}$ (Samples)	Reference-Region Gain (dB)	BER	CRC
FrFT baseline	45,755	2.78	N/A	Fail
Full proposed receiver	0	24.92	0	Pass

**Table 3:** Robustness under noise and INR sweeps.

Sweep	Value	Localization Error (Samples)	Cancellation Gain	BER	CRC
Receiver-noise level (dBFS)	−45	0	24.38 dB	0	Pass
	−40	0	24.38 dB	0	Pass
	−35	0	24.38 dB	0	Pass
	−30	0	24.38 dB	0	Pass
	−25	0	24.38 dB	0	Pass
Beat-domain INR (dB)	10	0	24.38 dB	0	Pass
	15	0	22.27 dB	0	Pass
	20	0	18.93 dB	N/A	Fail
	25	0	16.35 dB	N/A	Fail
	30	0	13.25 dB	N/A	Fail

**Table 4:** Recovery under identity and multipath communication channels.

Channel	Max Excess Delay	Localization Error	Residual Power Ratio	Cancellation Gain	CRC
Identity	0	0	0.0040	23.98 dB	Pass
Three-tap multipath	9	0	0.0038	24.20 dB	Pass

## Data Availability

The data presented in this study are available on request from the corresponding author.
